# Lifestyle Modification Program on a Metabolically Healthy Elderly Population with Overweight/Obesity, Young-Old vs. Old-Old. CONSEQUENCES of COVID-19 Lockdown in This Program

**DOI:** 10.3390/ijerph182211926

**Published:** 2021-11-13

**Authors:** Lidia Cobos-Palacios, Mónica Muñoz-Úbeda, Maria Isabel Ruiz-Moreno, Alberto Vilches-Perez, Antonio Vargas-Candela, Javier Benítez-Porres, Ana Navarro-Sanz, Maria Dolores Lopez-Carmona, Luis Miguel Pérez-Belmonte, Jaime Sanz-Canovas, Ricardo Gomez-Huelgas, Maria Rosa Bernal-Lopez

**Affiliations:** 1 Internal Medicine Department, Instituto de Investigación Biomédica de Málaga (IBIMA), Regional University Hospital of Málaga, University of Málaga, 29010 Málaga, Spain; cobospala-cios@gmail.com (L.C.-P.); monicamubeda@gmail.com (M.M.-Ú.); mruiz.salud@gmail.com (M.I.R.-M.); anto-nio.vargascandela@gmail.com (A.V.-C.); mdlcorreo@gmail.com (M.D.L.-C.); luismi-guelpb@hotmail.com (L.M.P.-B.); jaimesc25@hotmail.com (J.S.-C.); 2 Endocrinology and Nutrition Department, Instituto de Investigación Biomédica de Málaga (IBIMA), Virgen de la Victoria University Hospital, 29010 Málaga, Spain; alberto_v4@hotmail.com; 3 Physical Education and Sport Area, Faculty of Medicine, University of Málaga, 29016 Málaga, Spain; beni-tez@uma.es; 4 Sports Area, Sport Medicine, Málaga City Hall, 29006 Málaga, Spain; ansanz@malaga.eu

**Keywords:** SARS-CoV-2, metabolically healthy obesity, elderly population, young-old, old-old

## Abstract

The SARS-CoV-2 pandemic led to lockdowns, which affected the elderly, a high-risk group. Lockdown may lead to weight gain due to increased food intake and reduced physical activity (PA). Our study aimed to analyze the impact of a 12-month lifestyle intervention on a metabolically healthy overweight/obese elderly (MHOe) population and how the lockdown by COVID-19 affected this program. **Methods:** MHOe participants (65–87 years) were recruited to participate in a lifestyle modification intervention based on the Mediterranean diet (MedDiet) and regular PA. Participants were classified into two groups: young-old (<75 years) or old-old (≥75 years). Anthropometric and clinical characteristics, energy intake, and energy expenditure were analyzed at baseline and after 12 months of intervention. **Results:** The final sample included 158 MHOe participants of both sexes (age: 72.21 ± 5.04 years, BMI: 31.56 ± 3.82 kg/m^2^): 109 young-old (age: 69.26 ± 2.83 years, BMI: 32.0 ± 3.85 kg/m^2^) and 49 old-old (age: 78.06 ± 2.88 years, BMI: 30.67 ± 3.64 kg/m^2^). After 12 months of intervention and despite lockdown, the young-old group increased MedDiet adherence (+1 point), but both groups drastically decreased daily PA, especially old-old participants. Fat mass significantly declined in the total population and the young-old. Depression significantly increased (26.9% vs. 21.0%, *p* < 0.0001), especially in the old-old (36.7% vs. 22.0%, *p* < 0.0001). No significant changes were found in the glycemic or lipid profile. **Conclusions:** This study indicates that ongoing MedDiet intake and regular PA can be considered preventative treatment for metabolic diseases in MHOe subjects. However, mental health worsened during the study and should be addressed in elderly individuals.

## 1. Introduction

Obesity is a global health problem that affects individuals of all ages. Its prevalence has dramatically increased in recent decades. In most developed countries, a large percentage of individuals older than 65 years are overweight and the prevalence of obesity in this age group has risen to 20–30% [1,2]. Many elderly individuals reach old age already overweight and, worldwide, older adults represent the fastest growing population. Obesity in older adults is related to multiple chronic diseases, including type 2 diabetes mellitus (T2DM), hypertension, cardiovascular diseases, and certain types of cancer; these are the leading causes of death worldwide. In addition to these diseases, physiological changes due to aging, such as a decrease in bone mineral density and an increase in abdominal fat, increase the risk of non-fatal disability and a lower quality of life in this population [3,4]. In 2017, more than 41% of young-old individuals (aged 65–74 years) and more than 48% of old-old individuals (aged 75–84 years) in Spain were overweight [5] and more than 50% of young-old individuals and more than 40% of old-old individuals were obese [6].

Obesity is an important health issue, not only due to its high prevalence but also because of its morbidity and mortality. In 2017, over 4 million individuals died as a result of being obese or overweight according to the Global Burden of Disease report [7]. Obesity is normally associated with chronic and cardiovascular diseases, but there are some individuals who are highly resistant to developing obesity-related metabolic syndromes. This subset of obese patients is known as the metabolically healthy obese (MHO) [8]. Obesity and its associated diseases have been widely studied, but data on the MHO population are limited, especially on the MHO elderly (MHOe) population.

The main cause of obesity is an imbalance in calories consumed and calories expended, although other factors may be involved. Globally, there is a trend toward consuming high-energy foods that are usually high in free sugars and fat, as well as a trend toward performing less physical activity (PA). These trends are mainly caused by changes in social and environmental factors. Obesity is preventable and reversible and diet modifications along with regular PA can lead to weight loss. Reducing intake of foods that are high in free sugars and fat and increasing daily intake of fruits, vegetables, and legumes are healthy eating habits that can reduce the total energy (kcal) intake. The Mediterranean diet (MedDiet) is a dietary pattern characterized by a high consumption of fruits, vegetables, and legumes; olive oil as the main source of fat; moderate-to-high fish intake; moderate consumption of wine; and low consumption of meat and poultry [9]. MedDiet adherence is associated with a lower risk of developing T2DM and cardiovascular diseases [10,11].

In January 2020, the World Health Organization (WHO) announced an outbreak of a new coronavirus disease in Wuhan, China. It would eventually come to be known as coronavirus disease 2019 (COVID-19), which is caused by severe acute respiratory syndrome coronavirus 2 (SARS-CoV-2) infection. In March 2020, COVID-19 was declared a pandemic and affected countries worldwide. Its rapid, unpredictable spread, along with its potential severity, forced some countries to decree a lockdown of their population. Spain was one of the hardest hit countries and had one of the strictest, longest periods of restrictions. These prolonged lockdowns were a challenge to the physical and mental well-being of individuals of all ages [12], but they especially affected the elderly [13,14]. Older adults are more vulnerable to COVID-19 [15] and health authorities, politicians, and social media have widely reported this fact. In addition to the restrictions, fear of contracting COVID-19 led to decreased PA in older people [16].

Individuals with obesity are particularly vulnerable to COVID-19 and its complications [17]. A recent meta-analysis showed that excess visceral fat and high body mass index (BMI) are risk factors for severe COVID-19 [18] and put individuals at higher risk of requiring hospitalization and invasive mechanical ventilation. The mechanisms underlying this risk may be due to the fact that angiotensin-converting enzyme (ACE) 2 is the receptor for SARS-CoV-2 [19] and is expressed in many tissues, including white adipose tissue, which is more prevalent in visceral fat than in subcutaneous fat [20]. In addition to this mechanism, many individuals’ dietary habits changed during lockdown, with a higher frequency of food intake, snacking, and consumption of alcoholic beverages [21]; these habits were also observed in the elderly [22]. Unhealthy food habits, along with decreased PA due to lockdown, led to weight gain in many individuals, especially in people who were already obese [23].

This study aimed to explore the impact of a 12-month lifestyle modification program consisting of MedDiet and PA on the health status of a Spanish MHOe population (young-old and old-old). Due to the unique situation of the COVID-19 lockdown, participants’ social, psychological, and nutritional behaviors were analyzed, given the aforementioned links between obesity, the elderly, and COVID-19 restrictions.

## 2. Subjects and Methods

### 2.1. Study Design and Participants

This work involved an open cross-sectional study that included a MHOe population aged 65–87 years of both sexes. The inclusion criteria were elderly participants (≥65 years old) who were overweight/obese (BMI ≥ 28 kg/m^2^) with one or none of the following four cardiometabolic disorders [24]: (1) elevated blood pressure—systolic blood pressure ≥130 and/or diastolic blood pressure ≥85 mmHg (or antihypertensive drug treatment in a patient with a history of hypertension); (2) high triglyceride levels—≥150 mg/dL (or treatment with fibrates, nicotinic acid, or ω-3 fatty acids); (3) low high-density lipoprotein (HDL) cholesterol levels—<40 mg/dL in men or <50 mg/dL in women (or treatment with fibrates or nicotinic acid); and (4) high glucose levels—fasting plasma glucose ≥100 mg/dL (or a previous diagnosis of diabetes or treatment with antidiabetic drugs). The exclusion criteria were participants who had more than two of the aforementioned four disorders, those who were <65 years of age, those who had T2DM, or those had any metabolic disease. Participants were recruited from July 2019 to March 2020 at Healthy Aging Centers, which are part of the Sports Department (Sports Medicine) of the Málaga City Hall (Andalusia, Spain). Once possible participants were selected, investigators contacted them to inform them about the study and invite them to participate.

Deaths that occurred from 2018 to 2020 were verified via data from the Spanish Ministry of Health. Losses were defined as individuals who were not located after four attempts (including at least one attempt in the evening and one over the weekend), hospitalized individuals, and those who moved out of the city. Subjects who declined to answer the questionnaire were considered to have refused participation. When the refusal was voiced by telephone, the interviewer made one final attempt to include the patient by means of an in-person home visit.

### 2.2. Written Informed Consent

Selected subjects were invited to come to the Department of Internal Medicine at the Regional University Hospital of Málaga in order to inform them about the study design, orient them on the study’s main objectives, and request their written informed consent for voluntary participation. In the event they were unable to sign the form, a legal guardian was asked to sign.

### 2.3. Initial Visit

Subjects who wished to participate in the study had an initial visit with a nurse. Weight, height, BMI, waist circumference (WC), hip circumference, abdominal obesity (waist-to-hip ratio (WHR)), heart rate, and blood pressure measurements were taken. Weight was measured with an electronic scale (TANITA Body Composition Analyzer (TBF-300 MA) TANITA Corporation, 1–14–2 Maeno-cho, Itabashi-ku, Tokyo, Japan). Height was measured without shoes using a wall stadiometer (Stadiometer Barys Electra Model. 511-300-A0A ASIMED). BMI was calculated by dividing weight (kg) by height squared (m^2^). Obesity was defined as a BMI ≥ 30 kg/m^2^. WC was measured halfway between the last rib and the iliac crest using an anthropometric tape. Abdominal obesity was defined using the WC measurement and according to the WHO guidelines [25], with obesity in older males defined as a WC > 102 cm and in older females as a WC > 88 cm. WHO cutoff points for overall obesity were also used: individuals of both sexes with a BMI ≥ 30 kg/m^2^ were considered obese. Blood pressure (systolic/diastolic) was calculated as the mean of three measurements with a five-minute rest between them and was measured using an automated electronic sphygmomanometer (OMRON M7 (HEM-780-E), OMRON Healthcare Co. Ltd., Kyoto, Japan).

Blood samples were taken after a 12 h fast and biochemical measurements (triglycerides, HDL cholesterol, total cholesterol, LDL cholesterol, glycosylated hemoglobin, insulin, and glucose) were determined using routine methods at the Clinical Analysis Laboratory of the Regional University Hospital of Málaga. The Homeostatic Model Assessment of Insulin Resistance (HOMA-IR) was calculated as glucose (mg/dL) × insulin (µIU/mL)/405 [26]. Depression due to lockdown was measured in all participants by comparing scores on the geriatric depression scale (GDS) questionnaire completed at baseline and 12 months, after lockdown was declared [27,28]. Scores of 0–4 were considered normal and scores of ≥5 indicated presence of depression. A validated 14-item food frequency questionnaire was also completed by participants in order to determine MedDiet adherence at baseline and after 12 months of lifestyle intervention. High adherence was considered to be 12–14 points, moderate adherence 8–11 points, low adherence 5–7 points, and very low adherence <5 points [9].

### 2.4. Diet

Included subjects had an interview with a nutritionist. All subjects completed a non-consecutive, three-day dietary record (two workdays and one weekend day), which offered detailed information about the composition and cooking methods of all food consumed [29]. Subjects also completed a food frequency questionnaire [30] at the first visit and after 12 months of lifestyle modification. The MedDiet recommended to the study subjects included extra virgin olive oil, high amounts of fiber, and nuts. The recommended caloric intake was 1500–1750 kcal/day [31,32], with fat representing 30% (5–8% saturated fatty acids, 15–18% monounsaturated fatty acids, 5–8% polyunsaturated fatty acids, and <300 mg of cholesterol/day), carbohydrates representing 55% (<10% simple sugars, 40% complex sugars, and low glycemic index), and protein representing 15% of intake [32,33]. Once subjects’ eating habits were evaluated, the nutritionist and nurse provided guidance on lifestyle modifications.

### 2.5. Physical Activity (PA)

Subjects were informed of physical activity guidelines for individuals of their age. As part of the lifestyle intervention, participants performed regular PA with trained monitors (supervisors with a degree in the physical activity and sports sciences field) at the participants’ Healthy Aging Centers. In addition, we offered the possibility of attending an exercise program on Fridays at the Ciudad Jardín Sports Center, which is managed by the Málaga City Hall. This program was offered until the pandemic began. The sessions consisted of aerobic exercise, strength training, and exercises to improve flexibility and balance. In addition, participants kept a PA record using a GENEActiv Actigraph GT3X+ accelerometer to measure energy expenditure. It was used for one full week at the baseline visit and again at 12 months. During lockdown, a training video was sent to participants to offer them the possibility of continuing PA at home.

The body composition of participants’ whole body was measured via bone mineral density (BMD) using dual energy X-ray absorptiometry (DXA) (bone densitometer—BMD Hologic, Discovery QDR Series, Bedford, USA) at baseline and 12 months. Each subject was scanned by a certified technician, and the distinguished bone and soft tissue were calculated by computer algorithms (software version APEX 3.0, Hologic QDR 4500, Bedford, MA, USA). Calibration of the densitometer was checked daily against the standard calibration block supplied by the manufacturer (Phantom 21,965 Lumbar Spine with anthropomorphic characteristics of 4 hydroxyapatite vertebrae included in resin; coefficient of variation: 0.415%). To determine intertester reliability, two different observers manually selected the area for each subject [34]. The results indicated the percentage of body fat, fat mass, lean mass, total mass, and total fat.

### 2.6. Follow-Up Medical Visit

Participants who had any questions about the MedDiet or PA during the intervention or about any procedures had a telephone number they were able to call at any time. The follow-up medical visits were done 12 months after the start of the intervention. At the follow-up visit, the same protocol was followed as in the baseline visit: anthropometric measurements were taken, blood samples were analyzed, and questionnaires were completed in order to analyze the impact of the lifestyle intervention.

### 2.7. Statistical Analysis

Quantitative variables with a normal distribution were expressed as mean ± standard deviation (SD) and qualitative variables were expressed as percentages. Student’s *t*-test was used to compare quantitative variables and the chi-square test to compare qualitative variables. In order to calculate the sample size, we used the Simple Interactive Statistical Analysis (SISA) program. We based our calculations on previous MHO studies that demonstrated the cardiometabolic benefits of a MedDiet and PA when participants lost more than 5% their initial body weight [35,36]. The confidence level of the study was 95% (α error of 5%), statistical power was 80%, and a 5% loss rate was assumed. Therefore, a sample of 110 MHOe was required.

## 3. Results

**Figure 1:**
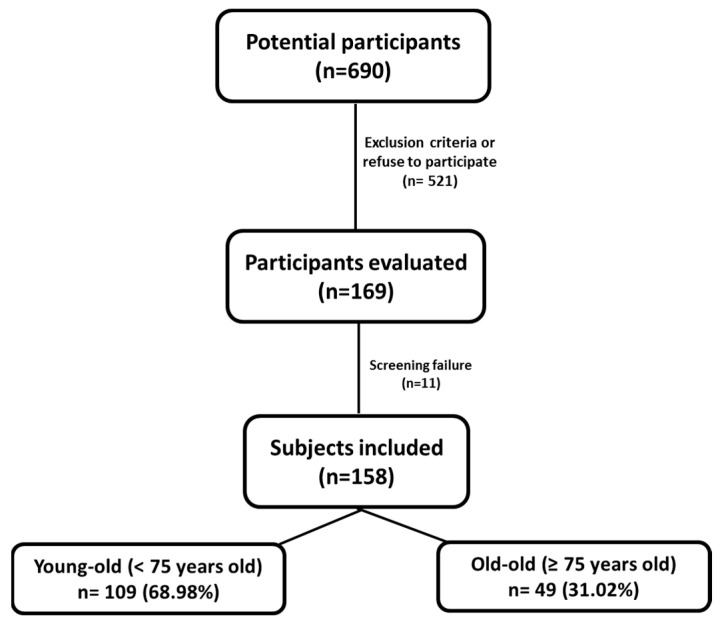
Screening and follow-up on the MHOe study population.

A total of 640 MHOe individuals were identified as possible participants. Of them, 169 came to the first visit regarding the intervention at the Regional University Hospital of Málaga. Eleven individuals refused to participate or met one or more of the exclusion criteria. Finally, 158 MHOe participants of both sexes (36 men (22.8%) and 122 (77.2%) women) were included in the study. Of them, 109 participants were <75 years old (young-old) and 49 participants were ≥75 years old (old-old) (Figure 1).

The mean age and BMI for all participants were 72.21 ± 5.04 years and 31.56 ± 3.82 kg/m^2^, respectively. For young-old MHOe, the figures were 69.26 ± 2.83 years and 32.0 ± 3.85 kg/m^2^ and for old-old MHOe, the figures were 78.06 ± 2.88 years and 30.67 ± 3.64 kg/m^2^. In regards to metabolic disorders, 76 (69.7%) young-old subjects had hypertension, 20 (18.3%) had high triglyceride levels, 2 (1.8%) men and 10 (9.2%) women had low HDL cholesterol levels, and 11 (10.1%) had high glucose levels. For old-old MHOe, 29 (59.2%) had hypertension, 6 (12.2%) had high triglyceride levels, 1 (2.0%) men and 6 (12.2%) women had low HDL cholesterol levels, and 7 (14.3%) had high glucose levels.

The anthropometric and clinical variables are summarized in Table 1. After 12 months of intervention, which was underway when lockdown was declared, there was no significant weight loss in the total population. Old-old participants did lose weight (−2.4 kg), but their BMI remained stable. WC decreased significantly in the total population (−3.5 cm), among the young-old (−3.5 cm), and among the old-old (−3.6 cm). In addition, the WHR decreased significantly in entire MHOe population and in both age groups (−0.2 and −0.1, respectively).

Participants showed moderate adherence to the MedDiet at baseline. After 12 months of intervention, the young-old group significantly improved adherence (9.5 ± 1.9 vs. 10.5 ± 1.4 points, respectively; *p* < 0.0001), but the old-old group maintained the same adherence (9.5 ± 2.0 vs. 9.8 ± 2.4 points, respectively, *p* = 0.34). The total study population significantly improved MedDiet adherence after 12 months of intervention (9.5 ± 2.0 vs. 10.3 ± 1.7 points, respectively; *p* < 0.0001). Energy intake results at baseline and after the intervention are shown in Table 2. A non-significant reduction in energy intake, carbohydrates, total proteins, polyunsaturated fatty acids, total fat, cholesterol, and vitamin D (VD) intake in the young-old population was observed after 12 months of intervention. However, old-old participants consumed +143.4 kcal/d and +34 g/d of carbohydrates after the intervention. The old-old group also consumed more fiber (+6.5 g/d) than the young-old group.

**Figure 2:**
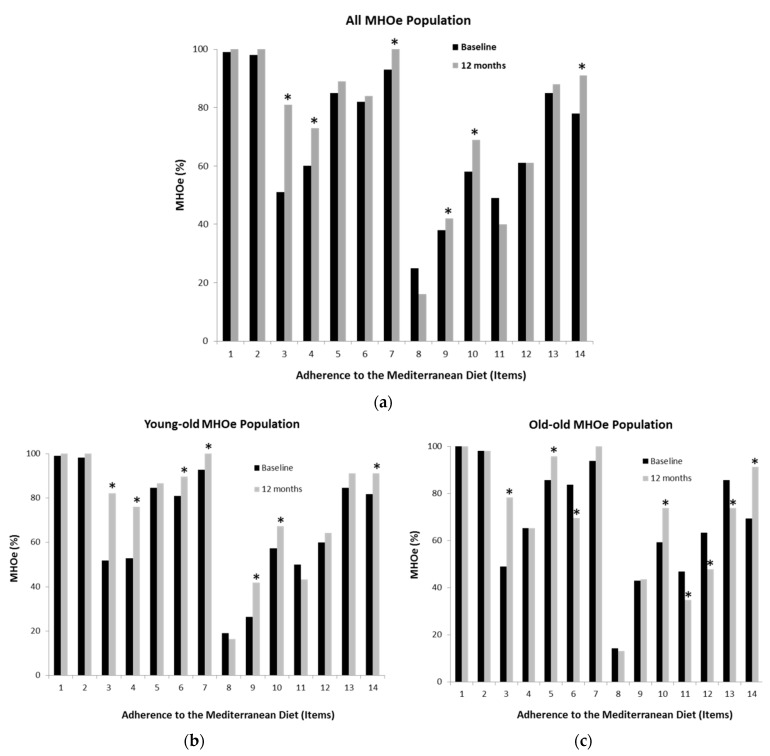
MedDiet adherence items at different periods of time for all studied subjects and classified by young-old and old-old participants. Patients completed a questionnaire on MedDiet adherence that asked about the items: 1. use of olive oil as the main cooking fat; 2. consumption of ≥2 tablespoons of olive oil per day; 3. consumption of ≥2 servings of vegetables per day; 4. consumption of ≥3 pieces of fruit per day; 5. consumption of <1 servings of red meat or sausages per day; 6. consumption of <1 servings of butter, margarine, or cream per day; 7. consumption of <1 carbonated or sugary drinks per day; 8. consumption of ≥3 glasses of wine per week; 9. consumption of ≥3 servings of legumes per week; 10. consumption of ≥3 servings of fish/seafood per week; 11. consumption of <3 servings of processed baked goods per week; 12. consumption of ≥1 servings of nuts per week; 13. preferential consumption of lean meats (chicken, turkey, rabbit); 14. consumption of home-cooked meals (cooked vegetables, pasta, rice, stir-fry) ≥2 times per week. * *p* ≤ 0.001.

A significant trend toward higher intake of olive oil, vegetables, fruit, legumes, fish, nuts, and lean meats after the intervention was observed in both the young-old and old-old MHOe population. Additionally, there was less consumption of red meat, butter, margarine, cream, carbonated and sugary drinks, and processed baked goods. In addition, participants ate more homemade meals each week (Figure 2). However, a significant increase in the intake of processed baked goods and a decrease in the intake of nuts or lean meats were observed among old-old participants.

After 12 months of the intervention, all participants were more sedentary, although the change was not statistically significant. Due to the lockdown, PA of any intensity drastically declined regardless of the participants’ age (Table 3). Body composition showed that in the total population and the young-old, fat mass significantly decreased after 12 months of intervention. In addition, total fat also decreased in population as a whole. Old-old participants had more lean mass (+0.5 kg) after 12 months of follow-up (Table 4).

After 12 months of intervention, 26.9% of total MHOe population presented with depression, a greater proportion than at the beginning of the study before lockdown (21.0%; *p* < 0.0001 with respect to baseline). The percentage of old-old participants with depression also increased significantly (22.0% vs. 36.7%; *p* < 0.0001). However, the percentage of young-old participants with depression remained nearly the same as at baseline (20.5% vs. 17.1%, respectively; *p* = 0.28).

The glycemic and lipid profiles are shown in Table 5. A slight yet significant increase in glucose levels was observed after the intervention in all MHOe participants, a change that was more notable among old-old participants (+1.9 mg/dL), though values remained within the normal range. However, this increase did not translate into higher glycosylated hemoglobin values. In fact, insulin and HOMA-IR were lower among these participants, but these changes were not statistically significant.

## 4. Discussion

Our study reveals the effect of a 12-month lifestyle intervention based on MedDiet consumption and regular PA in the MHOe population in our setting. Given that a lockdown was declared during the intervention, the elderly population faced challenges in maintaining a healthy lifestyle. The circumstances contributed to more cases of depression, especially among old-old participants. As other studies have demonstrated, our work shows that the mental and physical health of elderly individuals have been negatively affected by COVID-19 restrictions [37,38].

The SARS-CoV-2 pandemic is the major health crisis of our time and has the potential to cause devastating social, economic, and political consequences worldwide. In this context, it is important to study the social, psychological, nutritional, and PA behavior of the entire population, and especially of the populations with obesity, diabetes, or other metabolic diseases, who are more affected by COVID-19 and have seen increased mortality rates. It is essential to control obesity and overweight worldwide, especially during the pandemic and in the elderly population, in order to reduce not only obesity-related diseases, but also the gravity of consequences if these individuals do develop COVID-19. According to the World Obesity Federation, obesity-related conditions seem to worsen the effects of SARS-CoV-2 infection [39]. Evidence has shown that the renin–angiotensin–aldosterone system (RAAS), which contains the angiotensin-converting enzyme (ACE) 2 targeted by SARS-CoV-2, is involved in energy metabolism, food intake, inflammatory processes, oxidative stress, and blood pressure control, such that disease severity can increase with BMI [40]. Indeed, ACE 2 is largely expressed in adipose tissue, and significantly more so in visceral rather than peripheral subcutaneous adipose tissue [41].

Among the elderly, changes in appetite, eating behaviors, and body composition play an important role in the development of obesity. There is a physiological, age-related reduction in appetite known as the anorexia of aging [42]. It is a multifactorial condition caused by the reduction in energy expenditure that occurs in the elderly. Food choices change with age, with reduced consumption of high-protein foods observed among elderly adults. This is in line with our results, which showed that a MedDiet with high intake of olive oil and greater intake of vegetables, fruit, legumes, and fish, was consumed by the entire MHOe population, though old-old participants increased their consumption of processed baked goods and decreased their consumption of nuts and lean meats. Overall, the lifestyle intervention was quite successful despite Spain’s strict lockdown because participants continued consuming the MedDiet and homecooked meals and greater adherence to the MedDiet was observed, especially among old-old participants, with these data in concordance with this situation in Italy [43].

An important parameter that must also be analyzed and controlled in populations with obesity is VD level. Lockdowns led to reduced VD levels in populations worldwide, which influenced and aggravated the health condition of the population with obesity, who normally already have low levels of VD [44]. Furthermore, a normal VD level is extremely important for inducing cathelicidins and defensins to decrease the viral replication rate [45] and to modulate ACE-1 and ACE-2 expression, which leads to a protective effect on lipopolysaccharide-induced lung damage [46]. In Spain, there are long hours of sunlight, yet VD deficiency is notable in the entire population [47] and this was aggravated by lockdown. On the other hand, VD intake is typically deficient in elderly populations [48,49]. In this study, all participants had a non-significant decrease in VD intake at 12 months, irrespective of their age and gender, and this decline was greater in old-old participants. All participants also had very low intake of VD and after 12 months of intervention, the old-old participants had levels that were below baseline, data in concordance with other studies in Spain [50]. Analytical parameters, as well as the glycemic and lipid profile, remained in the normal range after 12 months of intervention in the entire MHOe population. Even slight changes in these parameters are enough to reduce cardiovascular risks associated with obesity.

During Spain’s lockdown, the level of PA among our population was lower than usual, in concordance with other studies [51]. Our MHOe population decreased their daily moderate–vigorous PA and increased sedentarism because going to sports centers was not permitted during lockdown. Furthermore, our population did not have the technological skills needed to download the PA videos that were provided to them. Lack of PA not only predisposes individuals to weight gain, but can also lead to the loss of strength, skeletal muscle mass, and immune competence [52]. MHOe subjects in our study were more sedentary and did less light, moderate, and moderate–vigorous PA (min/day) at 12 months of intervention compared to baseline. Although they had a more sedentary lifestyle due to the lockdown, the moderate–vigorous exercise they performed along with reduced intake of calories, fat, carbohydrates, protein, and cholesterol as part of a balanced MedDiet was enough to lead to weight loss and a reduced BMI and WC.

Aging leads to a change in body composition. On average, the elderly have more body fat and less lean mass than younger adults. There is also an increase in visceral fat due to redistribution from the subcutaneous to the intra-abdominal, intrahepatic, and intramuscular regions [53]. These changes are caused by decreased PA, reduced growth hormone secretion, and a lower basal metabolic rate, among other age-related factors. PA tends to decrease with age, especially in elderly adults with obesity, which further contributes to weight gain. Our MHOe population was very active in their daily lives, performing high intensity PA. Our results showed that in the total population and among the young-old, fat mass significantly decreased after 12 months of intervention. Lean mass increased among the old-old participants. This is in contrast with data that have demonstrated that functional capacity and mobility are significantly reduced in elderly adults with obesity [54] and that they are more likely to have joint pain, increased self-perception of functional limitations, more anxiety and depression, and are eventually more likely to be homebound [55]. Obesity exacerbates age-related physical dysfunction and is a strong predictor of frailty, disability, and poor quality of life among the elderly [56].

The elderly population with obesity has shown worsened psychological and physical abilities due to lockdown, especially in muscle strength, as a result of the increase in sedentarism [57]. Psychosocial burdens such as stress or depression are associated with obesity and this psychological support is essential to managing obesity [58]. During the COVID-19 pandemic, an increase in psychological distress was observed in infected individuals and in the general population in different countries [59,60]. Individuals suffering from obesity tend to be more vulnerable to distress due to the psychological impact of this disease; this distress was aggravated during COVID-19 lockdown, an aspect that must be analyzed in order to better manage obesity and its physical and psychological consequences. Psychological distress can also lead to emotional problems that may trigger binge eating in patients with obesity, which may be associated with poorer long-term outcomes [61]. This is in agreement with our data, which showed that after a 12-month lifestyle intervention, there were higher rates of depression in total MHOe population and among old-old participants.

## 5. Conclusions

Our results show that instilling healthy habits from childhood leads to healthy aging. To ensure healthy aging, it is necessary to follow a balanced diet and do regular PA, be it in a sport center, outside, or at home; these pillars of a healthy lifestyle are even more important in an exceptional situation such as lockdown. Accordingly, the prompt implementation of preventive health measures, such as promoting a healthy, active lifestyle among the elderly with obesity, emerges as a critical priority for public health policies that has become all the more relevant during the SARS-CoV-2 pandemic. The lockdown forced this population to remain at home, depriving them of regular PA practice and aggravating age-related complications. In addition, social, psychological, and nutritional problems increased among the elderly population, who are some of the most vulnerable members of our society.

## 6. Limits

This study did not have a control group. This cross-sectional study was conducted to evaluate the improvements caused by lifestyle modifications in a MHOe population, for which the study design could be considered as a limitation. However, the aim of this design was that each subject was their own control.

## Figures and Tables

**Table 1:** Anthropometric parameters at baseline and 12 months of the intervention in all subjects and classified by age range. Values are indicated as mean ± SD and the *p* value.

	Population	Baseline (B)	12 Months (12M)	*p* (B vs. 12M)
Body weight (Kg)	Young-Old	80.6 ±11.9	80.1 ± 12.2	0.27
	Old-Old	74.0 ± 9.6	71.6 ± 8.3	0.84
	All	78.5 ± 11.6	78.1 ± 11.9	0.29
BMI (Kg/m^2^)	Young-Old	31.9 ± 4.0	31.8 ± 4.4	0.36
	Old-Old	30.6 ± 3.7	30.4 ± 3.7	0.80
	All	31.5 ± 3.9	31.5 ± 4.3	0.35
WC (cm)	Young-Old	103.8 ± 8.9	100.3 ± 9.7	<0.0001
	Old-Old	99.9 ± 9.7	96.3 ± 9.9	<0.0001
	All	102.8 ± 9.2	99.3 ± 9.7	<0.0001
WHR	Young-Old	0.93 ± 0.08	0.92 ± 0.08	0.01
	Old-Old	0.92 ± 0.08	0.91 ± 0.09	0.03
	All	0.93 ± 0.08	0.91 ± 0.09	0.01
SBP (mmHg)	Young-Old	136.9 ± 19.4	135.0 ± 17.1	0.56
	Old-Old	137.6 ± 17.8	143.0 ± 20.2	0.52
	All	137.1 ± 18.9	137.2 ± 18.2	0.85
DBP (mmHg)	Young-Old	85.5 ± 11.1	84.3 ± 8.9	0.25
	Old-Old	82.8 ± 12.1	85.6 ± 8.7	0.86
	All	84.7 ± 11.4	84.6 ± 8.8	0.27
HR (ppm)	Young-Old	70.8 ± 10.8	69.4 ± 10.0	0.41
	Old-Old	69.3 ± 10.5	69.5 ± 13.2	0.56
	All	70.3 ± 10.7	69.5 ± 10.8	0.82

BMI: body mass index; WC: waist circumference; WHR: waist to-hip ratio; SBP: systolic blood pressure; DBP: diastolic blood pressure; HR: heart rate.

**Table 2:** Energy and food intake at baseline and after 12 months of intervention in the total study population and classified by age range. Values are indicated as mean ± SD and the *p* value.

	Population	Baseline (B)	12 Months (12M)	*p* (B vs. 12M)
Energy (Kcal/d)	Young-Old	1698.0 ± 397.3	1622.3 ± 53.6	0.24
	Old-Old	1688.2 ± 367.7	1831.6 ± 951.6	0.32
	All	1695.4 ± 388.1	1676.8 ± 574.0	0.92
Carbohydrates (g/d)	Young-Old	166.0 ± 71.1	150.6 ± 35.1	0.19
	Old-Old	162.4 ± 44.2	196.4 ± 62.2	0.29
	All	165.0 ± 64.9	162.5 ± 89.2	0.92
Total Proteins (g/d)	Young-Old	75.6 ± 15.7	71.7 ± 16.8	0.04
	Old-Old	74.1 ± 18.5	76.2 ± 28.3	0.39
	All	75.2 ± 16.4	72.9 ± 20.4	0.44
Total Fat (g/d)	Young-Old	79.6 ± 21.1	79.5 ± 20.5	0.90
	Old-Old	78.1 ± 18.9	82.0 ± 32.8	0.32
	All	79.2 ± 20.5	80.1 ± 24.2	0.47
SFA (g/d)	Young-Old	21.7 ± 7.3	22.7 ± 14.4	0.69
	Old-Old	21.0 ± 6.5	21.1 ± 6.3	0.88
	All	21.5 ± 7.1	22.3 ± 12.8	0.67
MUFA (g/d)	Young-Old	38.7 ± 9.7	39.9 ± 10.6	0.21
	Old-Old	37.8 ± 8.4	40.5 ± 16.0	0.20
	All	38.5 ± 9.3	40.1 ± 12.2	0.07
PUFA (g/d)	Young-Old	12.0 ± 5.7	11.3 ± 5.0	0.63
	Old-Old	12.1 ± 5.4	13.6 ± 10.0	0.08
	All	12.0 ± 5.6	11.9 ± 6.7	0.48
Cholesterol (mg/d)	Young-Old	324.7 ± 132.8	309.8 ± 114.4	0.02
	Old-Old	312.7 ± 96.8	311.3 ± 118.1	0.62
	All	321.5 ± 124.0	310.2 ± 114.7	0.06
Fiber (g/d)	Young-Old	18.8 ± 6.4	16.8 ± 6.1	0.03
	Old-Old	18.9 ± 7.6	25.4 ± 9.3	0.23
	All	18.9 ± 6.7	19.0 ± 6.1	0.77
Vitamin D (µg/d)	Young-Old	3.5 ± 3.0	3.5 ± 3.9	0.81
	Old-Old	3.2 ± 3.1	2.3 ± 1.8	0.17
	All	3.4 ± 3.0	3.2 ± 3.5	0.75

SFA: saturated fatty acids; MUFA: monounsaturated fatty acids; PUFA; polyunsaturated fatty acids.

**Table 3:** Energy expenditure as measured using a GENEActiv Actigraph GT3X+ accelerometer at baseline and after 12 months of intervention in the total study population and classified by age range. Values are indicated as mean ± SD and the *p* value.

	Population	Baseline (B)	12 Months (12 M)	*p* (B vs. 12 M)
Sedentarism (min/day)	Young-Old	702.7 ± 134.1	731.5 ± 133.5	0.21
	Old-Old	762.1 ± 166.6	774.7 ± 156.8	0.20
	All	718.7 ± 145.3	742.8 ± 140.5	0.07
Physical Activity				
Light PA (min/day)	Young-Old	706.4 ± 157.4	268.6 ± 68.2	<0.0001
	Old-Old	772.1 ± 152.6	234.5 ± 101.0	<0.0001
	All	726.6 ± 158.4	259.7 ± 79.0	<0.0001
Moderate PA (min/day)	Young-Old	282.6 ± 85.3	54.3 ± 38.2	<0.0001
	Old-Old	251.4 ± 95.2	24.7 ± 29.9	<0.0001
	All	273.0 ± 89.3	46.6 ± 38.4	<0.0001
Vigorous PA (min/day)	Young-Old	67.8 ± 50.8	4.5 ± 7.0	<0.0001
	Old-Old	33.5 ± 34.1	1.5 ± 3.8	<0.0001
	All	57.2 ± 48.8	3.7 ± 6.5	<0.0001
Moderate-Vigorous PA (min/day)	Young-Old	357.6 ± 120.6	327.4 ± 97.3	<0.0001
	Old-Old	287.5 ± 117.9	260.8 ± 116.3	0.06
	All	336.0 ± 123.7	310.0 ± 106.2	<0.0001

**Table 4:** Body composition as measured by a bone densitometer (DXA) at baseline and after 12 months of intervention in the total study population and classified by age range. Values are indicated as mean ± SD and the *p* value.

	Population	Baseline (B)	12 Months (12 M)	*p* (B vs. 12 M)
Fat Mass (Kg)	Young-Old	33.2 ± 8.0	31.8 ± 8.9	0.04
	Old-Old	30.7 ± 5.5	30.5 ± 5.7	0.63
	All	32.6 ± 7.5	31.5 ± 8.2	0.03
Lean Mass (Kg)	Young-Old	48.0 ± 9.4	47.3 ± 11.7	0.47
	Old-Old	42.0 ± 5.7	42.5 ± 6.6	0.29
	All	46.6 ± 9.0	46.2 ± 10.9	0.59
Total Mass (Kg)	Young-Old	81.2 ± 12.2	79.2 ± 16.8	0.17
	Old-Old	72.7 ± 8.7	73.0 ± 9.7	0.61
	All	79.1 ± 11.9	77.7 ± 15.6	0.20
Total Fat (%)	Young-Old	40.8 ± 7.6	39.5 ± 9.0	0.07
	Old-Old	42.1 ± 4.9	41.7 ± 4.9	0.34
	All	41.1 ± 7.0	40.0 ± 8.3	0.05

**Table 5:** Analytical parameter at baseline and after 12 months of follow-up in all MHOe populations and classified by age range. Values are indicated as mean ± SD and the *p* value.

	Population	Baseline (B)	12 Months (12M)	*p* (B vs. 12M)
Glucose (mg/dL)	Young-Old	87.1 ± 19.7	87.5 ± 10.4	0.04
	Old-Old	87.2 ± 13.4	89.1 ± 13.2	0.05
	All	87.1 ± 17.9	88.0 ± 11.2	0.01
Glycosylated Hemoglobin (%)	Young-Old	5.7 ± 0.6	5.7 ± 0.3	0.01
	Old-Old	5.7 ± 0.3	5.6 ± 0.3	0.81
	All	5.7 ± 0.5	5.7 ± 0.3	0.01
Insulin (mU/L)	Young-Old	12.4 ± 7.4	12.7 ± 8.2	0.26
	Old-Old	9.6 ± 5.1	8.9 ± 3.8	0.84
	All	11.5 ± 6.9	11.7 ± 7.4	0.39
HOMA-IR	Young-Old	2.8 ± 2.1	2.8 ± 1.8	0.27
	Old-Old	2.1 ± 1.3	1.9 ± 0.9	0.93
	All	2.6 ± 1.9	2.6 ± 1.7	0.36
Total Cholesterol (mg/dL)	Young-Old	212.9 ± 33.5	207.6 ± 33.8	0.06
	Old-Old	204.6 ± 31.0	210.5 ± 32.0	0.84
	All	210.2 ± 32.8	208.4 ± 33.2	0.09
HDL-c (mg/dL)	Young-Old	59.5 ± 14.4	61.1 ± 16.7	0.23
	Old-Old	60.5 ± 13.0	68.8 ± 25.1	0.21
	All	59.8 ± 13.9	63.2 ± 19.5	0.84
LDL-c (mg/dL)	Young-Old	129.8 ± 29.7	124.5 ± 32.2	0.02
	Old-Old	122.3 ± 27.0	126.4 ± 26.9	0.77
	All	127.4 ± 29.0	125.0 ± 30.7	0.04
Triglycerides (mg/dL)	Young-Old	106.0 [87.0–135.0]	103.0 [80.0–132.0]	1.00
	Old-Old	99.0 [72.5–129.0]	101.0 [84.0–122.0]	0.42
	All	103.0 [80.0–132.0]	107.0 [83.0–137.0]	0.65

## Data Availability

Not applicable.
